# Spiritual quality of life and spiritual coping: evidence for a two-factor structure of the WHOQOL spirituality, religiousness, and personal beliefs module

**DOI:** 10.1186/s12955-015-0212-x

**Published:** 2015-02-25

**Authors:** Christian U Krägeloh, D Rex Billington, Marcus A Henning, Penny Pei Minn Chai

**Affiliations:** Department of Psychology, Auckland University of Technology, Private Bag 92006, Auckland, 1142 New Zealand; University of Auckland, Auckland, New Zealand

**Keywords:** Quality of life, WHOQOL-SRPB, Coping, Spiritual wellbeing, Confirmatory factor analysis

## Abstract

**Background:**

The WHOQOL-SRPB has been a useful module to measure aspects of QOL related to spirituality, religiousness, and personal beliefs, but recent research has pointed to potential problems with its proposed factor structure. Three of the eight facets of the WHOQOL-SRPB have been identified as potentially different from the others, and to date only a limited number of factor analyses of the instrument have been published.

**Methods:**

Analyses were conducted using data from a sample of 679 university students who had completed the WHOQOL-BREF quality of life questionnaire, the WHOQOL-SRPB module, the Perceived Stress scale, and the Brief COPE coping strategies questionnaire. Informed by these analyses, confirmatory factor analyses suitable for ordinal-level data explored the potential for a two-factor solution as opposed to the originally proposed one-factor solution.

**Results:**

The facets WHOQOL-SRPB facets *connected*, *strength*, and *faith* were highly correlated with each other as well as with the religious coping sub-scale of the Brief COPE. Combining these three facets to one factor in a two-factor solution for the WHOQOL-SRPB yielded superior goodness-of-fit indices compared to the original one-factor solution.

**Conclusions:**

A two-factor solution for the WHOQOL-SRPB is more tenable, in which three of the eight WHOQOL-SRPB facets group together as a spiritual coping factor and the remaining facets form a factor of spiritual quality of life. While discarding the facets *connectedness*, *strength*, and *faith* without additional research would be premature, users of the scale need to be aware of this alternative two-factor structure, and may wish to analyze scores using this structure.

## Introduction

The importance of spirituality and existential concerns in health care settings has been firmly established within the biopsychosociospiritual model [1], and is now increasingly recognized beyond its initially limited applications with terminally ill and older patients [2]. A number of questionnaires are available that measure various aspects of spirituality or spiritual wellbeing [3], including the *Spiritual Well-being Scale* (SWBS) [4], the *Functional Assessment of Chronic Illness Therapy-Spiritual Well-being* (FACIT-Sp) [5], or the SpREUK (German acronym for *Spiritual and Religious Attitudes in Dealing with Illness*) [6]. In their systematic review, Monod et al. [7] identified 35 instruments that measure some aspects of spirituality in clinical research, and they proposed a classification system that distinguishes between measures of general spirituality, spiritual wellbeing, spiritual needs, and spiritual coping.

Spirituality, religiousness, and existential concerns have also become a major component of health-related quality of life (HRQOL) [8] and thus part of so-called patient-reported outcome measures [9,10]. The World Health Organization Quality of Life (WHOQOL) tools provide a particularly attractive suite of HRQOL instruments due to its original development as part of international collaborations spanning across 15 centers in 14 countries and its ability to claim strong cross-cultural validity [11]. During the development of its HRQOL instruments, the WHOQOL Group recognized the importance of spirituality to HRQOL [12], which was subsequently included in the WHOQOL-100 questionnaire as a domain alongside *physical*, *psychological*, *levels of independence*, *social relationships*, and *environmental* QOL [11]. In the 26-item abbreviated version of the questionnaire, the WHOQOL-BREF, spirituality is no longer a stand-alone domain, but one item about existential considerations (*meaningful life*) was carried over into the psychological domain [13]. To enable more detailed investigations of spirituality and QOL, the WHOQOL-SRPB was later developed using the same international collaborative methodology of the original WHOQOL [14]. This questionnaire module contains eight facets of spirituality, religiousness, and personal beliefs (*connectedness to a spiritual being or force*, *meaning of life*, *awe*, *wholeness and integration*, *spiritual strength*, *inner peace/serenity/harmony*, *hope and optimism*, and *faith*) expressed by four items each. Items are worded in ways that do not make any particular assumptions and are thus applicable to individuals with a range of different spiritual, religious, and personal beliefs.

The article reporting on the development of the 32-item WHOQOL-SRPB module [14] only reported preliminary results of the factor structure of the instrument. Using an exploratory factor analysis, an eight-factor solution for the module was proposed, with each facet being a separate factor. When testing their French translation of the WHOQOL-SRPB, Mandhouj et al. [15] were not able to replicate this factor structure, with the largest deviation being that *connectedness to a spiritual being or force* and *faith* loaded together as one factor. At that stage, concerns with the instrument’s conceptual clarity had already been raised: Moreira-Almeida and Koenig [16] argued that the three facets *faith*, *connectedness to a spiritual being or force*, and *spiritual strength* are different from the other facets in that they appear to reflect coping strategies rather than spiritual wellbeing. As with Mandhouj et al. [15], Krägeloh et al. [17] found that *faith*, *connectedness to a spiritual being or force*, and *spiritual strength* were highly correlated, and this high collinearity prevented *faith* and *connectedness to a spiritual being or force* from being entered as predictors in a multiple linear regression. Unlike the other facets that were positive predictors of WHOQOL-BREF domains in this regression analysis, *spiritual strength* produced significant negative associations, further highlighting that this facet may be conceptually different from the others [17].

The only study so far [18] reporting on results from a confirmatory factor analysis conducted with the WHOQOL-SRPB [14] module also reported some potential deviations from its original structure. When testing a six-factor model by adding items of the WHOQOL-SRPB module to the spirituality domain of the WHOQOL-100, excellent fit indices were obtained. However, based on a preceding exploratory factor analysis, the facets *hope* and *inner peace* were not included. While the authors [18] concluded that spiritual QOL made a significant independent contribution to overall QOL, they also raised the possibility of multidimensionality of spiritual QOL.

Very recently, an abbreviated version of the WHOQOL-SRPB (so-called WHOQOL-SRPB BREF) has been developed [19]. The WHOQOL-SRPB was shortened by selecting the most suitable item from each of the eight SRPB facets and the one spirituality item (*meaning in life*) located in the psychological domain of the WHOQOL-BREF. An exploratory factor analysis of these nine items revealed two factors, with *faith*, *connectedness to a spiritual being or force*, *spiritual strength*, and *wholeness and integration* loading together. When conducting an exploratory factor analysis of the nine SRPB items together with the WHOQOL-BREF, the authors observed some deviations from the established factor structure of the WHOQOL-BREF, but concluded that a five-factor solution was tenable in which the nine SRPB items form a fifth domain alongside the four WHOQOL-BREF domains of *physical*, *psychological*, *social*, and *environmental QOL*.

Clearly, more detailed investigations of the factor structure of the WHOQOL-SRPB are needed, particularly using confirmatory factor analysis. The purpose of the present study was to provide such an investigation, particularly of the suggestion that this measure of spiritual QOL may be multidimensional [18] and may contain one factor that could be more accurately described as spiritual coping [17]. The dataset by Chai et al. [20] was suitable for this purpose since participants had completed the WHOQOL-BREF, WHOQOL-SRPB, as well as the Brief COPE as a measure of coping strategies [21] and the Perceived Stress Scale (PSS) [22]. Using this sample of 679 university students, we used confirmatory factor analysis appropriate for ordinal-level data to explore the potential for an alternative two-factor solution for the WHOQOL-SRPB module.

## Methods

### Participants

The present study investigated the psychometric properties of the WHOQOL-SRPB using a dataset that had previously been published as an article on the relationships between QOL, coping, and spirituality, religiousness, and personal beliefs [20]. This dataset was from a sample of 679 university students in New Zealand and was suitable for the present purposes as it contained measures from the WHOQOL-SRPB [14], the Brief COPE questionnaire [21], the PSS [22], and the New Zealand version of the WHOQOL-BREF [23], also validated for use with students [24]. Detailed demographic information about the sample was reported by Chai et al. [20]. As the data were obtained from university students, the average age of the participants was relatively low (M = 22.83, SD = 6.88). Around 73% of the students were female students, and approximately half of the participants reported being affiliated with a religious faith.

### Instruments

#### WHOQOL-BREF

The 26-item WHOQOL-BREF questionnaire is available as a validated New Zealand version [23] and has also been validated for use in medical students [24]. All items are scored on a five-point Likert scale, and missing items were imputed by the rounded average of the other items of the same domain, but only if less than half of the items in that domain were missing. Two items measure global QOL and health, and the remaining 24 items are part of one of the following four domains: physical QOL (seven items), psychological QOL (six items), social relationships (three items), and environmental QOL (eight items). The psychological domain of the WHOQOL-BREF contains one item from the spiritual QOL domain of the WHOQOL-100 [13]. For the present purposes of comparing previously proposed WHOQOL-BREF factor structures with alternatives, this item was assigned to the spiritual QOL domain, consistent with previous approaches [19]. The number of items in the psychological domain therefore decreased to five.

#### WHOQOL-SRPB

The 32-item WHOQOL-SRPB module [14] contains eight facets of spirituality, religiousness, and personal beliefs (*connectedness to a spiritual being or force*, *meaning of life*, *awe*, *wholeness and integration*, *spiritual strength*, *inner peace/serenity/harmony*, *hope and optimism*, and *faith*) that are worded in ways that are considered acceptable to participants of a wide range of religious and nonreligious beliefs [2].

#### Brief COPE

This 28-item questionnaire measures 14 different adaptive and maladaptive dispositional coping strategies, expressed by two items each [21]. These include *active coping*, *planning*, *positive reframing*, *acceptance*, *humor*, *religion*, *using emotional support*, *using instrumental support*, *self-distraction*, *denial*, *venting*, *substance use*, *behavioral disengagement*, and *self-blame*. The first eight coping strategies are generally considered as adaptive, and the remaining six as maladaptive. However, the factor structure of the questionnaire is unstable, and a wide variety of higher-order factor structures have been proposed [25]. The present study analyzed scores of the individual strategies by summing the score of the two items of each strategy, without proposing any higher-order structure. While the Brief COPE is typically administered using a four-point Likert scale, Chai et al. [20] used a five-point scale instead. Missing data were not imputed for the Brief COPE, which means that no sub-scales scores were calculated when at least one item on that sub-scale was missing.

#### PSS

This 14-item questionnaire inquires about the stress level perceived by the respondent during the past month [22]. A summary score was calculated yielding an overall level of perceived stress.

### Data analysis

Confirmatory factor analyses were conducted using LISREL v. 8.80, and all remaining data analyses with IBM SPSS v. 22.0. Two types of confirmatory factor analyses were conducted. Firstly, the factor structure of the 32-item WHOQOL-SRPB module was evaluated by comparing the simplest factor solution (with all facets being a separate factor which in turn load onto a higher-order *spiritual QOL* factor) versus an alternative model that was informed by the analyses of the Chai et al. [20] dataset outlined below. These analyses explored whether postulating a second higher-order factor (*spiritual coping*) may be a tenable alternative to be tested by confirmatory factor analysis. Secondly, the five-factor solution of the WHOQOL-BREF (with *spiritual QOL* as a fifth domain) proposed by Skevington et al. [19] was compared to alternative models that were also based on the analyses conducted on the Chai et al. [20] dataset.

As the data were ordinal in nature, confirmatory factor analyses used an asymptotically distribution free (ADF) method with polychoric correlations and asymptotic co-variance matrices [26,27]. For small to moderate sample sizes, the method of diagonally weighted least squares is recommended as a suitable ADF method [27], and was therefore also selected for the present study. Error variances were not allowed to be correlated. Since chi-square values tend to become inflated with increases in sample size [28], model fits were evaluated using a set of goodness-of-fit indices: root mean square error of approximation (RMSEA), comparative fit index (CFI) and standardized root mean square residual (SRMR). Following the frequently quoted guidelines by Hu and Bentler [29], model fits were considered acceptable if RMSEA < 0.06, CFI > 0.90, and SRMR < 0.08.

## Results

Table 1 shows the correlations of the eight WHOQOL-SRPB facets with each other and the global QOL item. All SRPB facet scores were significantly correlated with each other, typically with values of *rho* around .50 or .60. The three facets *connectedness*, *strength*, and *faith* were highly correlated with each other, with correlation coefficients exceeding .80. None of these three facets were correlated with the global QOL item. The remaining items were significantly correlated with global QOL, and correlation coefficients ranged from .16 to .30.

**Table 1:** **Spearman’s** ***rho*** **correlation coefficients for the overall QOL item from the WHOQOL-BREF and the eight facet scores of the WHOQOL-SRPB**

	**Overall QOL**	**Connectedness**	**Meaning of life**	**Awe**	**Wholeness**	**Strength**	**Inner peace**	**Hope**
Connectedness	-.07	-						
Meaning of life	.17**	.52**	-					
Awe	.24**	.46**	.60**	-				
Wholeness	.16**	.53**	.58**	.61**	-			
Strength	.01	.84**	.61**	.59**	.68**	-		
Inner peace	.24**	.37**	.49**	.50**	.66**	.51**	-	
Hope	.30**	.28**	.56**	.61**	.61**	.44**	.65**	-
Faith	-.05	.82**	.57**	.45**	.61**	.85**	.48**	.38**

**p < .01.

The following analysis explored the contribution of each facet to overall QOL, while also controlling for perceived stress. Using a hierarchical linear regression, the demographic variables *age* and *gender* were entered in the first block, followed by the facet scores and perceived stress in the second block. Due to issues of collinearity, the facets *connectedness* and *faith* were not entered. *Meaning of life*, *wholeness*, and *inner peace* did not significantly predict overall QOL (Table 2). *Awe*, *inner peace*, and *hope* were positive predictors of overall QOL, but *strength* and *perceived stress* presented with a negative associations.

**Table 2:** **Results from a hierarchical multiple-linear regression (unstandardized coefficient** ***B*** **and standardized coefficient** β**) with the overall QOL item from the WHOQOL-BREF as the outcome variable and with age and gender as predictor variables in the first block, followed by the WHOQOL-SRPB facets and the PSS summary score as predictors in the second block**

	***B***	**β**
Age	-.00	-.01
Gender	.15	.09*
Meaning of life	.05	.06
Awe	.09	.11*
Wholeness	-.03	-.04
Strength	-.11	-.17**
Inner peace	.02	.03
Hope	.19	.20**
Perceived stress	-.03	-.28**

*p < .05, **p < .01.

Due to high collinearity, *connectedness* and *faith* were not entered. The total proportion of variance explained (*r* ^2^) in Block 2 was .20.

Table 3 presents correlations of all WHOQOL-SRPB facets with the coping strategies scores of the Brief COPE. With the exception of *humor*, adaptive coping strategies were generally significantly correlated with the SRPB facet scores. Most of these correlations were relatively small, only occasionally exceeding .30. The coping strategy *religion*, in contrast, showed high correlations with *connectedness*, *strength*, and *faith* (>.70), moderate correlations with *meaning of life* and *wholeness* (>.40), and small to moderate correlations with *awe*, *inner peace*, and *hope* (>.20). The relationship between SRPB facets and maladaptive coping strategies was less clear, with generally small and negative correlations.

**Table 3:** **Spearman’s** ***rho*** **correlation coefficients for the Brief COPE sub-scale scores and the eight facet scores of the WHOQOL-SRPB**

	**Connectedness**	**Meaning of life**	**Awe**	**Wholeness**	**Strength**	**Inner peace**	**Hope**	**Faith**
Active coping	.13**	.28**	.26**	.28**	.19**	.22**	.28**	.16**
Planning	.14**	.24**	.22**	.20**	.18**	.08*	.15**	.15**
Positive reframing	.19**	.32**	.30**	.33**	.23**	.25**	.34**	.21**
Acceptance	.09*	.22**	.17**	.22**	.13**	.19**	.25**	.10**
Humor	-.04	.02	.04	.07	-.04	.05	.08*	-.04
Religion	.81**	.43**	.38**	.48**	.74**	.35**	.23**	.76**
Emotional support	.26**	.27**	.25**	.28**	.25**	.21**	.16**	.26**
Instrumental support	.17**	.19**	.14**	.19**	.17**	.13**	.09*	.15**
Self-distraction	.06	-.06	-.06	-.04	-.02	-.09*	-.13**	.02
Denial	-.20**	-.26**	-.16**	-.17**	-.21**	-.18**	-.19**	-.21**
Venting	.11	-.15**	-.16**	-.07	.04	-.06	-.24**	.09*
Substance use	-.02	-.25**	-.24**	-.16**	-.09*	-.18**	-.29**	-.02
Behavioral disengagement	.16**	-.04	.01	.00	.12**	-.11**	-.15**	.13**
Self-blame	-.01	-.17**	-.14**	-.15**	-.08*	-.31**	-.28**	-.04

*p < .05, **p < .01.

**Figure 1:**
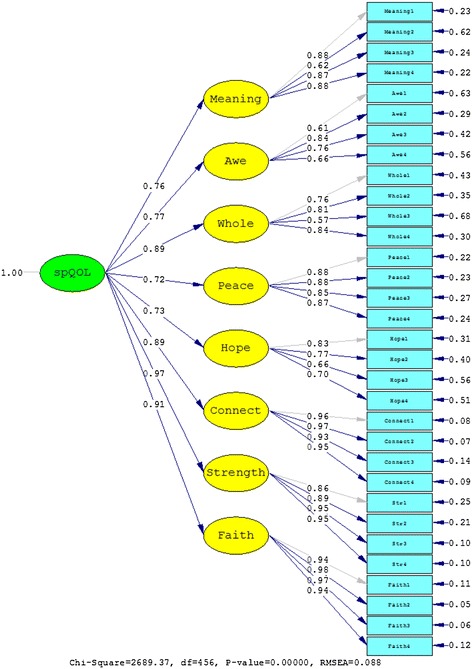
**Results from fitting the data to Model 1.**

**Figure 2:**
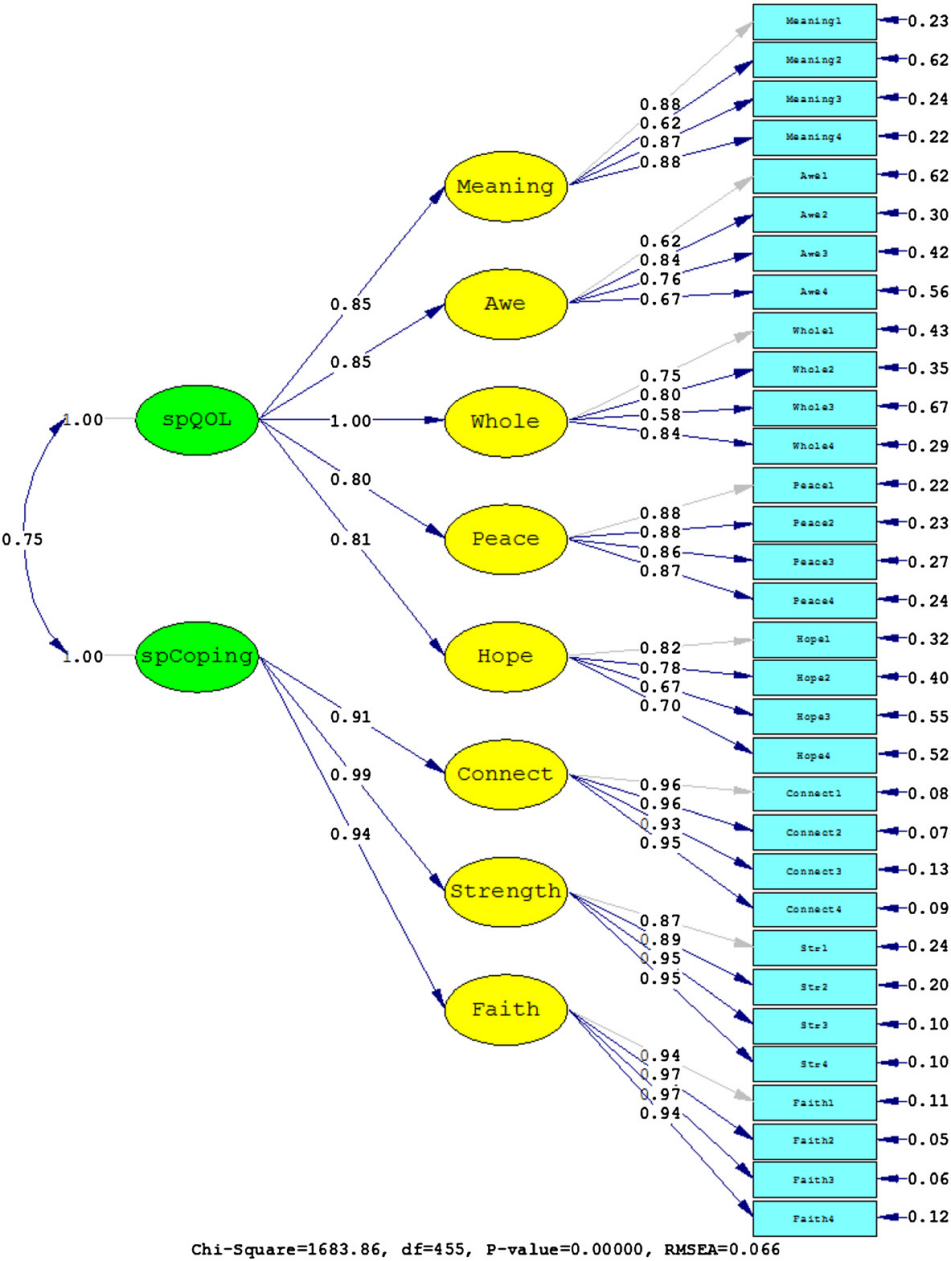
**Results from fitting the data to Model 2.**

The first confirmatory factor analyses tested the factor structure of the WHOQOL-SRPB module alone, in which all facets were part of one higher-order spiritual QOL factor (Model 1). Informed by the preceding analyses that correlated SRPB items with coping measures, an alternative factor solution (Model 2) was tested which subsumed the three facets *connectedness*, *strength*, and *faith* under one higher-order factor (spiritual coping) and the remaining ones under another one (spiritual QOL). Table 4 shows a summary of the good-of-fit indices for the two alternative models. The improvement of the nested model with two higher-order factors compared to the model with one higher-order factor was significant (χ^2^(1) = 1005.51, *p* < .01). Values of RMSEA, CFI, and SRMR also indicated strongly that the solution with two higher-order factors was superior. The results of fitting the data to Models 1 and 2 are also shown in Figures 1 and 2, respectively.

**Table 4:** **Goodness-of-fit indicators of alternative models: Satorra-Bentler scaled** χ^**2**^ **, RMSEA, CFI, and SRMR**

	**df**	**χ** ^**2**^	**RMSEA**	**CFI**	**SRMR**
Model 1	456	2689.37	0.088	0.974	0.107
Model 2	455	1683.86	0.066	0.986	0.075
Model A	459	2892.14	0.092	0.908	0.099
Model B	372	1247.06	0.061	0.962	0.065
Model C	458	1642.55	0.064	0.955	0.087

Values of RMSEA, CFI, and SRMR are shown with three decimal places.

**Figure 3:**
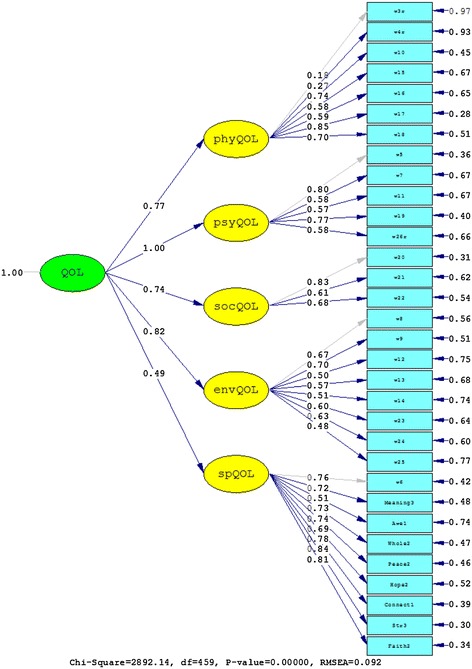
**Results from fitting the data to Model A.**

**Figure 4:**
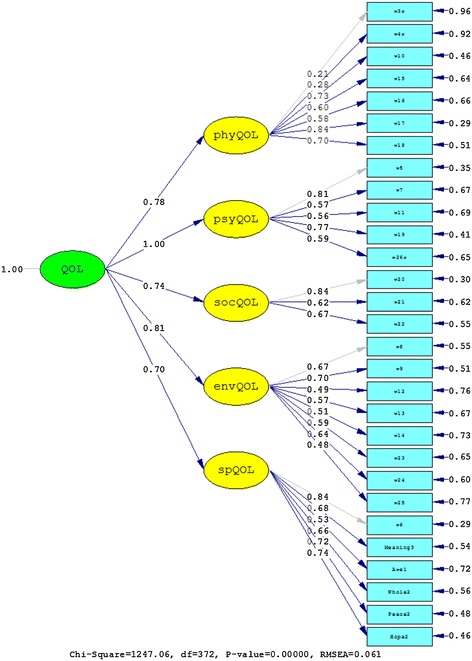
**Results from fitting the data to Model B.**

**Figure 5:**
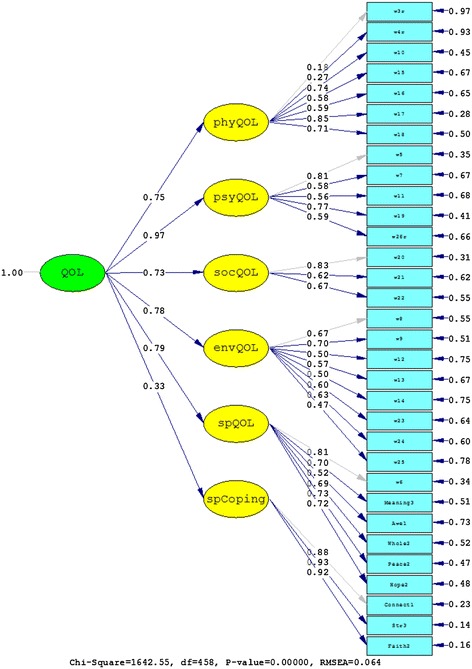
**Results from fitting the data to Model C.**

The second confirmatory factor analyses tested the five-factor structure of the WHOQOL-BREF (Model A) with one item from the psychological domain and one item from each of the eight facets of WHOQOL-SRPB module (*meaning of life*, *awe*, *wholeness and integration*, *inner peace/serenity/harmony*, and *hope and optimism*) forming a fifth domain alongside physical QOL, psychological QOL, social relationships, and environmental QOL of the WHOQOL-BREF. This structure was proposed by Skevington et al. [19] for the WHOQOL-SRPB BREF, the abbreviated version of the WHOQOL-SRPB. Fits were compared to Model B that had the same five-factor structure but that did not contain the three SRPB facets *connectedness*, *strength*, and *faith*. These facet items were not included in Model B as they appeared to be items that were more related to coping. In the six-factor structure of Model C, these three facet items were included but were grouped into an additional separate spiritual coping factor (*connectedness*, *strength*, and *faith*) alongside the five factors of Model B. Both Model B and Model C exhibited clearly improved fit indices compared to Model A (Table 4), and the nested Model C provided a significantly better fit than Model A (χ^2^(1)=1249.59, *p*<.01). The results of fitting the data to Models A, B and C are also shown in Figures 3, 4, and 5, respectively. Note the low factor loadings for the WHOQOL-BREF items 3 (*being free of pain*) and 4 (*free of dependence on medicine and treatment*) exhibited low factor loadings.

## Discussion

The present study adds important new empirical evidence to the limited formal investigations that have been conducted so far on the factor structure of the WHOQOL-SRPB. The article on the original development of the instrument reported results from a preliminary exploratory factor analysis where the four items from each facet loaded together as separate factors [14]. As these analyses were conducted on a global dataset of more than 5,000 participants in 18 countries around the world and collected using standard WHOQOL procedures, isolated reports [15] from individual countries about deviations from this factor structure may be of relatively limited concern. However, the fact that deviations from the factor structure had emerged repeatedly and were generally driven by similar facets [15,19] highlighted the need for further investigation. The present study confirmed previous hypotheses [16,17] that the facets *connectedness*, *strength*, and *faith* are more accurately described as *spiritual coping* than *spiritual QOL*. Unlike the other WHOQOL-BREF facets, these three facets were particularly highly correlated with the religious coping sub-scale of the Brief COPE [21]. Grouping these three facets together as a separate factor also resulted in more tenable solutions in the confirmatory factor analyses.

Considerable debate has centered around the distinctions between various aspects of spirituality and measures purporting to assess the role of spirituality in health care settings [30]. If the WHOQOL-SRPB is used as a measure of spirituality, correlations with wellbeing will be tautological, as items refer to positive affect and positive human traits such as altruistic values and activities [16,30]. However, the WHOQOL-SRPB was never intended to be a measure of spirituality. The original authors of the scale [14] described it as spirituality, religiousness, and personal beliefs as they relate to HRQOL, and Skevington et al. [19] explicitly used the expression *spiritual QOL* to capture the aspects of the items in that module. In the classification system proposed by Monod et al. [7], the WHOQOL-SRPB is recognized as a measure of spiritual wellbeing. Results from factor analyses clearly place the SRPB items as a separate domain of HRQOL [18,19], and the present study confirms these findings.

In a measure of spiritual QOL, then, do facets of spiritual coping (*connectedness*, *strength*, and *faith*) have a role to play or does their inclusion diminish the conceptual clarity of the WHOQOL-SRPB? In other words, are these facets best to be dropped from the WHOQOL-SRPB such as in Model B or should they to be retained as a separate factor such as in Model C? The principles of parsimony and simplicity favor Model B that excludes the facets *connectedness*, *strength*, and *faith* as they are more related to coping rather than QOL. The results from the confirmatory factor analyses indicate that the precision of the instrument is also improved that way. However, arguments can also be made for retaining these facets. First of all, extensive and thoroughly conducted focus group work around the world repeatedly highlighted these facets as important to spiritual QOL for participants with a wide range of spiritual, religious, and personal beliefs [2,14,19]. *Connectedness*, in particular, is considered a core element of spirituality [30], and the issues with this facet thus appear to be related more to the way it is worded. One avenue for future research may thus be attempting to re-word the items from the *connectedness*, *strength*, and *faith* facets so that they express more clearly aspects of spiritual wellbeing rather than coping. On the other hand, items inquiring about spiritual coping may be a means to capture spiritual distress—an aspect typically not covered in measures of spiritual wellbeing [7]. This may increase the precision of the instrument [7] and acknowledge the fact that spirituality and spiritual QOL may not be associated exclusively with adaptive aspects of coping but equally with unsuccessful and maladaptive coping strategies [31].

A limitation of the present study is that analyses were conducted on a dataset from a sample of university students. Certain model misfits such as the low factor loadings for the WHOQOL-BREF items 3 (*being free of pain*) and 4 (*free of dependence on medicine and treatment*) obtained here and similar problems with these items reported elsewhere [24,32] appear to be related to the collection of this type of information from samples with predominantly young people. Future work will need to confirm these findings with participants of a wider age range and with both healthy and ill participants, as is typically the case in WHOQOL work [12,14]. Furthermore, the present study inquired about participants’ dispositional coping strategies. Rather than assessing coping strategies in response to acute stress, assessment of dispositional coping relies on participants reflecting back on how they generally cope with stress, which is likely to be affected by retrospective bias.

To conclude, the present study adds to the limited number of studies that have formally investigated the factor structure of the WHOQOL-SRPB. Analyses confirmed previous hypotheses that the three facets *connectedness*, *strength*, and *faith* tap into a slightly different concept than the remaining five facets of the WHOQOL-SRPB. Patterns of correlations with measures of coping strategies and subsequent confirmatory factor analyses revealed that these three facets may be better described as facets of spiritual coping. However, discarding the facets *connectedness*, *strength*, and *faith* without additional research would be premature. The extensive focus group work during the development of the WHOQOL-SRPB noted the importance of these facets to QOL [14], and future research may thus attempt to re-word these facets so that they are expressed as spiritual QOL rather than spiritual coping. In the mean time, users of the scale need to be aware of its alternative two-factor structure, and may even wish to analyze scores in this manner, depending on circumstances of application. While inclusion of *connectedness*, *strength*, and *faith* has advantages in terms of content validity, precision of the instrument is improved if they are removed or analyzed as a separate domain.

## Abbreviations

QOL: Quality of life
WHOQOL: World Health Organization Quality of Life
SRPB: Spirituality, Religiousness, and Personal Beliefs
CFA: Confirmatory factor analysis
